# Large carnivores under assault in Alaska

**DOI:** 10.1371/journal.pbio.3000090

**Published:** 2019-01-15

**Authors:** William J. Ripple, Sterling D. Miller, John W. Schoen, Sanford P. Rabinowitch

**Affiliations:** 1 Global Trophic Cascades Program, Department of Forest Ecosystems and Society, Oregon State University, Corvallis, Oregon, United States of America; 2 Alaska Department of Fish and Game, Anchorage, Alaska, United States of America; 3 United States National Park Service, Anchorage, Alaska, United States of America

## Abstract

In Alaska, gray wolves (*Canis lupis*), brown bears (*Ursus arctos*), and black bears (*U*. *americanus*) are managed in most of the state in ways intended to significantly reduce their abundance in the expectation of increasing hunter harvests of ungulates. To our knowledge, Alaska is unique in the world because this management priority is both widespread and mandated by state law. Large carnivore management in Alaska is a reversion to outdated management concepts and occurs without effective monitoring programs designed to scientifically evaluate impacts on predator populations. Large carnivore management in Alaska should be based on rigorous science including the status and trends of carnivore populations.

## Abstract 2

This Perspective article argues that current large carnivore management in Alaska is a reversion to short-sighted and discredited concepts of the past and occurs without monitoring programs designed to scientifically evaluate the impacts on predator populations.

When Aldo Leopold saw “a fierce green fire dying” in the eyes of a gray wolf he’d just shot, he recognized that his actions taken in the hope of creating a “hunters’ paradise” of deer was ill conceived [1]. Most of the world now recognizes that apex predators have great intrinsic value as well as providing vitally important ecosystem services. In many cases, these services outweigh some of the inconveniences to humans associated with large carnivore populations [2]. At an accelerating rate during recent decades in Alaska, however, brown bears (Fig 1), black bears, and gray wolves have been targeted for significant reductions in abundance in the expectation this will result in more wild ungulates (moose [*Alces alces*], caribou [*Rangifer tarandus*], and deer [*Odocoileus hemonius sitkensis*]) available for hunter harvest. A management priority favoring wild ungulates over large carnivores acquired the force of law with the passage in 1994 of Alaska’s Intensive Management Law. This law effectively mandates management to reduce large carnivores and increase human harvests of wild ungulates. The Alaska Intensive Management efforts are occurring without rigorously collected data on the impacts of these management practices on large carnivores [3,4,5] and ecosystems [e.g., 2].

**Fig 1:**
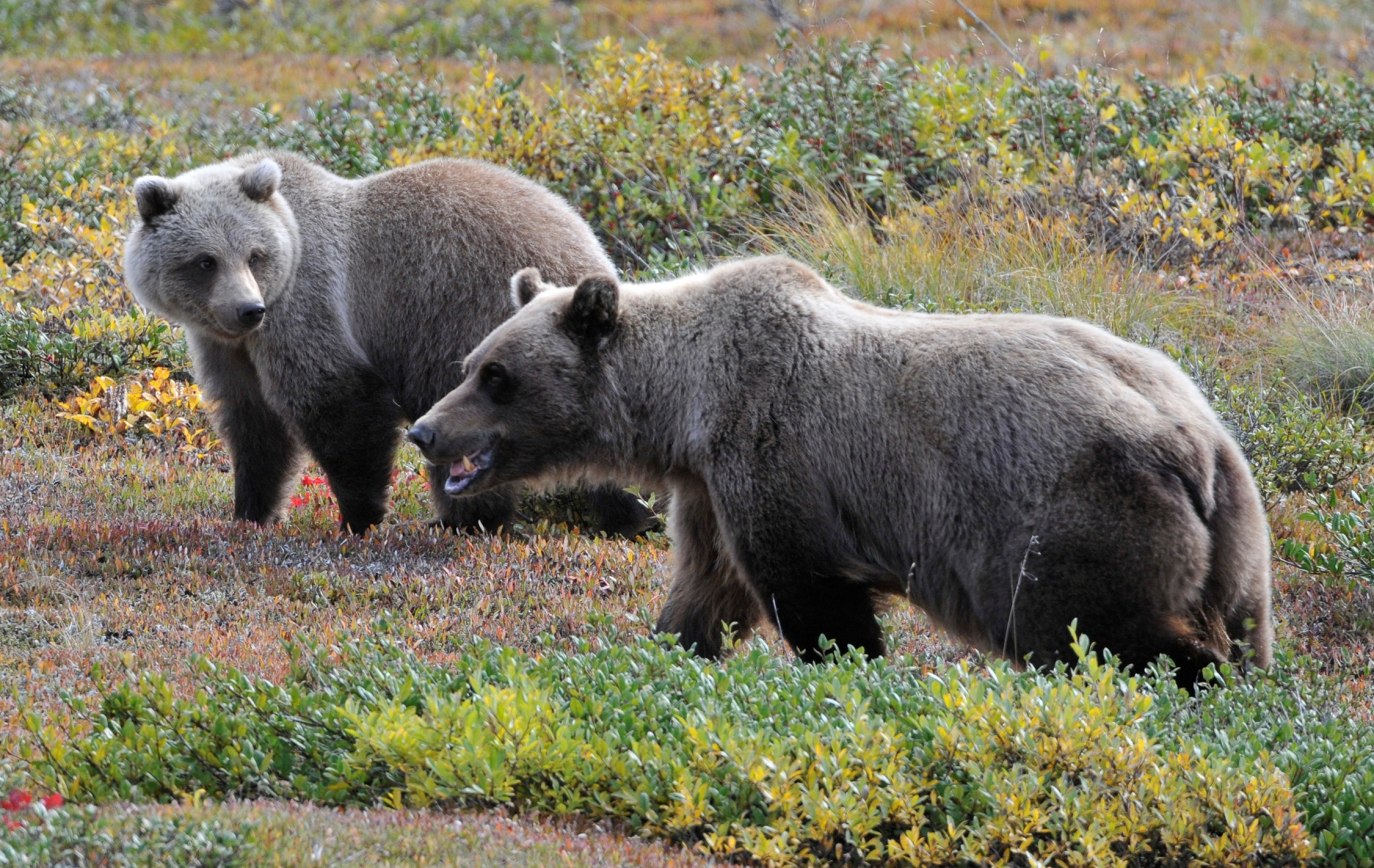
Alaskan brown bears in Denali National Park. Photo credit: J.W. Schoen.

Historically, gray wolves and brown bears were nearly extirpated in the conterminous United States because of persecution and habitat loss [6]. Similar reductions occurred throughout the world (e.g., [7] for Europe]. In Alaska, large areas of intact habitat for large carnivores persist, although in some areas, habitats and populations are depleted by human activities such as roads, logging, mining, and other development.

The Alaska Intensive Management law sets a management priority for high levels of harvests of wild ungulates in areas where these ungulates are “important for human consumption” (Alaska Statutes 16.02.255). The law specifies that Intensive Management must occur when the Alaska Board of Game makes a finding that the harvestable numbers of ungulates are insufficient to meet human demand for game meat. Under this law, before the Board can change hunting regulations to reduce human take of ungulate species, Intensive Management must occur. Although habitat management such as controlled burns to create early-succession moose habitat is an identified Intensive Management technique under the law, the most significant Intensive Management efforts have been implemented by liberalizing hunting regulations for large carnivores [3 for brown bears]. The lack of significant Intensive Management efforts from habitat improvement is precluded by scale, cost, and in the case of fire, threats to human structures [8]. In some places, termed Predator Control Areas, especially aggressive efforts include agency shooting of bears (both species) from helicopters, snaring of bears, and shooting female brown bears accompanied by cubs [3]. By 2017, the last remaining Predator Control Area for bears was eliminated. In wolf Predator Control Areas, allowed and utilized techniques include shooting of wolves by agency staff from helicopters, land and aerial hunting by the public, and carbon monoxide poisoning of pups in dens. Alaska conveniently defines “predator control” efforts as occurring only in these Predator Control Areas; this allows the state to claim that “predator control” is ongoing in only a small portion of Alaska. However, the degree that Intensive Management is accomplished by liberalized general hunting regulations for large carnivores is far more geographically extensive than the Predator Control Areas. For brown bears, regulation liberalizations include techniques such as shooting bears in dens, baiting bears, long (sometimes year-round) open hunting seasons, elimination of resident tag fees, and liberalized individual harvest quotas of 2 per year [3]. In a state of about 1,509,600 km^2^, 91% has been identified by the Alaska Board of Game as being important for human consumption of ungulates and therefore eligible for Intensive Management actions for one or more of the three wild ungulate species (compiled from 5 Alaska Administrative Code 92.108). Of this, the largest portion (60.1% of Alaska) is for moose (Fig 2).

**Fig 2:**
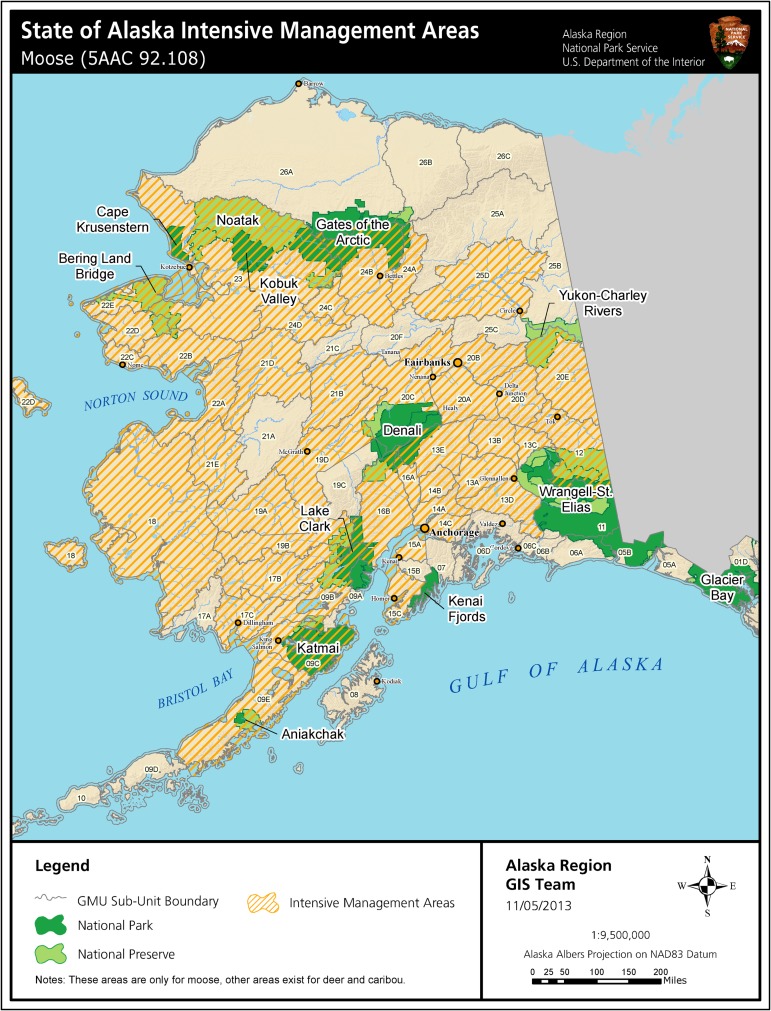
Areas in Alaska authorized for Intensive Management of large carnivores to benefit moose hunters (shown as crosshatched). Predator reduction (bears and/or wolves) occurs in essentially all of these authorized areas through liberalized hunting regulations for large carnivores. Additional areas are similarly identified for caribou (including Unit 26 in the far north) and deer (southeast Alaska through Prince William Sound and Kodiak Island). *Source*: *US National Park Service*.

Intensive Management regulations for all three species of large carnivores have been liberalized in increasing proportions of the state over the last 30 years. Reported kills of brown bears by hunters more than doubled during the last 30 years in a liberalized brown bear hunting area comprising 76% of the state [3]. Major liberalizations of hunting regulations for black bears and wolves also occurred, including expanded bag limits and extensions of seasons into times of the year when hides have little value.

Even Alaska’s 11 National Preserves managed by the United States National Park Service are not refugia from predator reduction regulations adopted by the state of Alaska. This is because a provision in the 1980 Alaska National Interest Lands Conservation Act allows sport hunting in Alaskan National Preserves, and the state determines who qualifies as a sport hunter (normally all Alaska residents and often nonresidents). This means that Alaska’s predator hunting regulations generally apply on National Preserves even though this is inconsistent with National Park Service policy guidelines, which state:

“The Service does not engage in activities to reduce the numbers of native species for the purpose of increasing the numbers of harvested species (i.e., predator control) …” ([9] Section 4.4.3).

Since about 2010, the National Park Service in Alaska has resisted adopting some of the most extreme of the state’s predator-reduction hunting and trapping regulations in National Preserves. The National Park Service currently has the legal authority to do this (Federal Register, 80 FR 205, 23 October 2015). The federal administration that took office in 2017, however, is currently proposing to reverse this 2015 rule, thereby constraining the ability of the National Park Service to resist adopting Alaska’s liberal hunting and trapping regulations on National Preserves (80 Federal Register 64325, RIN 1024-AE38). Similarly, nationwide, the current administration is also attempting to require that the U.S. Fish and Wildlife Service attempt to “align” hunting and trapping regulations on National Wildlife Refuges with regulations of the encompassing state. This constrains the ability of national wildlife refuge managers to manage hunting in ways consistent with national interests. In Alaska, this proposal makes it difficult for national wildlife refuge managers to resist adopting the state’s predator control regulations such as baiting for brown bears on the Kenai National Wildlife Refuge [3]. If successful in forcing managers of National Wildlife Refuges to adopt state hunting and trapping regulations, national priorities for wildlife management within refuges could be compromised throughout the United States.

Recent ecological studies have demonstrated the fundamental importance of apex predators in stabilizing ecosystems [2,10,11]. The removal or significant reduction of large carnivores can trigger a chain of events that can create a downward spiral toward ecosystem simplification [10]. Because research strongly suggests that apex predators regulate ecosystem structure and function, it would make sense for wildlife managers to carefully evaluate the ecological role of apex predators prior to implementing programs designed to reduce large carnivores. Hunting, especially selective hunting for large trophy animals, also can have adverse behavioral and genetic consequences for hunted populations [12,13]. A recent study concluded that Alaska’s wolf predator control management, adjacent to the Yukon–Charley Rivers National Preserve, had adverse impacts on wolf populations within the national preserve where there was no wolf control [14].

Science-based management of large carnivores in most of Alaska will require the political will and wisdom to repeal Alaska’s Intensive Management law. Alternatively or additionally, it will require professional wildlife managers to resist adoption of predator reduction regulations that are not conducted as experiments and/or do not include adequate monitoring programs of both carnivores and ungulates; this was a key recommendation in the 1997 report of National Research Council [5]. Furthermore, in Alaska and other states, the U.S. Department of the Interior needs to meet its legal mandate to manage for natural and healthy ecosystems in ways that are in the national interest. In Alaska, this will require not aligning hunting and trapping regulations on National Park Preserves and National Wildlife Refuges with state regulations that are designed to reduce naturally occurring densities of large carnivores. The state of Alaska also should be candid with the public about the absence of science supporting the efficacy of predator control programs to achieve established objectives with regard to ungulate harvests instead of making unsupported claims of “success” for wolf reduction efforts in publicly distributed booklets about Intensive Management (e.g., [15]). For bears, there are not even any claimed successes for increased harvests of adult moose or caribou resulting from increased bear harvests [3]. Appointments by the Alaska Governor to the Alaska Board of Game, which sets Alaska hunting regulations, should include members who recognize the importance and value of large carnivores both to ecosystem function [2] as well as to the state’s economy and wildlife viewing enthusiasts [16]. Mechanisms and funding must be in place to ensure science-based management that includes adequate monitoring and research of predator–prey relationships and trends [3,5,17]. Information campaigns and other grass roots efforts by concerned citizens and nongovernmental organizations are likely needed to remedy current unsound management practices for large carnivores in Alaska.

Alaska is unique in the world as a place where brown bear, black bear, and gray wolf populations are intentionally targeted for population reductions in efforts to increase human harvests of wild ungulate prey species and this priority is mandated by state statute. Similar management priorities do not occur in Europe [7,18]. The situation in Europe, however, is complicated by the private ownership of wildlife in many countries, which creates economic incentives to control predators from landowners who lease hunting rights. Predators are controlled in some other areas of the world to reduce losses of domestic livestock. In parts of Canada, there are concerns that reductions in wolf abundance may be necessary to bolster woodland caribou populations depleted by habitat losses [19].

In most of the world, there has been a paradigm shift moving away from predator control to supposedly benefit ungulates [20]. Enlightened scientific management at the scale of ecosystems is needed to put Alaska back on the path to avoiding the errors in predator management experienced a century ago when Leopold first noticed the fierce green fire fading in the eyes of a dying wolf.
