# Correction: Large carnivores under assault in Alaska

**DOI:** 10.1371/journal.pbio.3000282

**Published:** 2019-05-22

**Authors:** William J. Ripple, Sterling D. Miller, John W. Schoen, Sanford P. Rabinowitch

The Alaska Department of Fish and Game has asked *PLOS Biology* to clarify that the affiliation ‘Alaska Department of Fish and Game’ listed for co-authors Sterling D. Miller and John W. Schoen are for their former affiliations with that agency, prior to retirement 20 years ago, and that the viewpoints expressed in the article do not reflect the position of the Alaska Department of Fish and Game.

Similarly, co-author Sanford P. Rabinowitch is retired from the United States National Park Service and his co-authorship does not reflect the position of that agency.

The author byline and affiliations should read as follows:

William J. Ripple^1^, Sterling D. Miller^2,¤a^, John W. Schoen^2,¤b^, Sanford P. Rabinowitch^3,¤b^

**1** Global Trophic Cascades Program, Department of Forest Ecosystems and Society, Oregon State University, Corvallis, Oregon, United States of America, **2** Alaska Department of Fish and Game (ret.), Anchorage, Alaska, United States of America, **3** United States National Park Service (ret.), Anchorage, Alaska, United States of America

¤a Current address: Missoula, Montana, United States of America

¤b Current address: Anchorage, Alaska, United States of America
